# Ventricular Tachycardia Crisis: Assessing Norepinephrine vs. Stress Steroids in the Battle Against Waterhouse-Friderichsen Syndrome With Distributive Shock

**DOI:** 10.7759/cureus.55093

**Published:** 2024-02-27

**Authors:** Iyad Y Idries, Iryna Zadoretska, Anna Nevolina, Melissa Andrade, Rebecca Galer, Vijay Jaswani, Moshe Gunsburg

**Affiliations:** 1 Internal Medicine, Brookdale University Hospital Medical Center, Brooklyn, USA; 2 Hematology and Oncology, Institute of Blood Pathology and Transfusion Medicine of the National Academy of Medical Sciences of Ukraine, lviv, UKR; 3 Emergency Medicine, Brookdale University Hospital Medical Center, Brooklyn, USA; 4 Radiology, One Brooklyn Health-Interfaith Medical Center, Brooklyn, USA; 5 Electrophysiology, Brookdale University Hospital Medical Center, Brooklyn, USA

**Keywords:** distributive shock, stress steroids, adrenal hemorrhage, meningococcal infection, adrenal insufficiency, ventricular tachycardia, waterhouse-friderichsen syndrome

## Abstract

Waterhouse-Friderichsen syndrome (WFS) is a rare but life-threatening complication associated with acute hemorrhagic necrosis of the adrenal glands, primarily linked to meningococcal infection. This report details the case of a 62-year-old female with HIV/AIDS and substance misuse who presented with ventricular tachycardia and hemodynamic instability. Subsequent evaluation revealed WFS in the context of disseminated meningococcal infection. The case highlights the diversity of WFS manifestations and the diagnostic challenges, particularly in patients with comorbidities. Managing WFS involves a delicate balance of steroids and vasopressors, necessitating a multidisciplinary approach. Timely diagnosis and intervention are critical in mitigating the high mortality associated with this syndrome.

## Introduction

Waterhouse-Friderichsen syndrome (WFS) is a rare and life-threatening condition marked by shock, petechial rash, and bilateral adrenal hemorrhages resulting in acute adrenal failure [1]. While Neisseria meningitidis commonly presents as meningitis (80-85% cases) [2], WFS can manifest in diverse forms, posing diagnostic challenges. Our case involves a 62-year-old female with HIV/AIDS and substance misuse, presenting with ventricular tachycardia and hemodynamic instability. The evaluation revealed disseminated meningococcal infection and subsequent diagnosis of WFS. This case sheds light on the complexities of diagnosing and managing WFS, especially in individuals with comorbidities, highlighting the need for early detection and appropriate therapeutic interventions involving steroids and vasopressors. The report further explores the association of cardiac arrhythmias with sepsis, emphasizing the impact of aggressive resuscitation, imaging, antibiotic administration, and corticosteroid replacement on patient outcomes.

## Case presentation

A 62-year-old female with a past medical history of diabetes mellitus, asthma, HIV/AIDS with a CD4 count of 494, and substance use disorder presented with dizziness at a methadone clinic. On-site, Emergency Medical Services found the patient hypotensive with MAP <65, leading to her admission to the Emergency Department. She remained hypotensive despite fluid resuscitation of 30 cc/kg and exhibited tachycardia and tachypnea. Additional complaints included nausea, vomiting, and chronic neck and back pain, which typically responded to methadone.

Clinical examination revealed worsening mentation, prompting endotracheal intubation for airway protection, on cardiac monitor wide complex tachycardia suggestive of ventricular tachycardia that persisted after electrical cardioversion attempts; however, it resolved following the administration of 2 g of magnesium sulfate and 150 mg of intravenous amiodarone bolus. Subsequently, the patient was initiated on an amiodarone drip of 1 mg/kg for six hours followed by 0.5 mg/kg and maintained on norepinephrine for hemodynamic support.

Notably, lab findings included a lactic acid of 10.10 mmol/L and a white blood cell count of 26.3 x10^3/uL with a left shift as seen in Table 1. She was then admitted to the medical intensive care unit for presumed septic shock, suspected secondary to acute cholecystitis.

**Table 1:** Lab findings WBC: White Blood Cell Count, RBC: Red Blood Cell Count, HGB or HBG: Hemoglobin, HCT: Hematocrit, MCV: Mean Corpuscular Volume, MCH: Mean Corpuscular Hemoglobin, MCHC: Mean Corpuscular Hemoglobin Concentration, RDW: Red Cell Distribution Width, MPV: Mean Platelet Volume, PT: Prothrombin Time, INR: International Normalized Ratio, PTT: Partial Thromboplastin Time, BUN: Blood Urea Nitrogen, AST: Aspartate Aminotransferase, ALT: Alanine Aminotransferase, CO_2_: Carbon Dioxide

Test	Result	Reference value
Complete Blood Count		
WBC	26.3 10x3/uL	4.5 - 11.0 10x3/uL
RBC	4.3810x6/uL	3.95 - 4.83 10x6/uL
HBG	14.2 g/dL	11.4 - 15.5 g/dL
HCT	42.3 %	37 - 43.7 %
MCV	96.8 fL	82 - 94.5 fL
MCH	32.5 pg	26.9 - 32.5 pg
MCHC	33.6 g/dL	31.9 - 35.0 g/dL
RDW	15.5 %	12.6 - 14.9 %
MPV	9.7 fL	7.9 - 10.6 fL
Platelets	121 10x3/uL	180 - 401 10x3/uL
Neutrophils Auto	93.2 %	47.9 - 74.1 %
Lymphocytes Auto	3.2 %	17.8 - 41.1 %
Monocytes Auto	3.5 %	4.5 - 10.7 %
Eosinophils Auto	0.0 %	0.1 - 4.7 %
Basophils Auto.	0.1 %	0.3 - 1.1 %
Neutrophils Absolute	24.60 10x3/uL	2.30 - 6.80 10x3/uL
Lymphocytes Absolute	0.8 10x3/uL	1.30 - 3.00 10x3/uL
Basophils Absolute	0 10x3/uL	0.00 - 0.10 10x3/uL
Coagulation Panel		
PT	18.7 sec	9.8 - 13.4 sec
INR	1.66	0.85 - 1.15
PTT	42.5	24.9 - 35.9 sec
Complete Metabolic Panel		
Glucose	299 mg/dL	70 - 99 mg/dL
BUN	17 mg/dL	7 - 25 mg/dL
Creatinine	1.1 mg/dL	0.6 - 1.2 mg/dL
Sodium	135 mEq/L	136 - 145 mEq/L
Potassium	4.6 mEq/L	3.5 - 5.1 mEq/L
Chloride	99 mEq/L	98-107 mEq/L
CO_2_	16 mEq/L	21 - 31 mEq/L
Calcium	7.6 mg/dL	8.6 - 10.3 mg/dL
Anion Gap	20 mEq/L	8.00 - 12.00 mEq/L
Phosphorus	3.4	
Protein total	5.4 g/dL	6.4 - 8.9 g/dL
Albumin	3.0 g/dL	3.5 - 5.7 g/dL
Bilirubin total	2.6 mg/dL	0.3 - 1.0 mg/dL
AST	49 U/L	13 - 39 U/L
ALT	44 U/L	7 - 52 U/L
Blood gas analysis		
PH	7.11	
pCO_2_, arterial	56.8 mmHg	35.0 - 45.0 mmHg
pO_2_, arterial	85.3 mmHg	80.0 - 110.0 mmHg
HCO_3_, arterial	18.0 mmol/L	22.0 - 26.0 mmol/L
O2sat, arterial	92.4	95.0 - 100.0 %
Ionized Ca	4.3 mg/dL	4.5 - 5.3 mg/dL
Carboxyhemoglobin	1.2 %	0.0 - 1.5 %

Subsequent evaluations with ultrasonography showed minor pericholecystic fluid, gallbladder wall thickening, and a gallstone. Despite these findings, surgical intervention was ruled out given computed tomography (CT) of the abdomen and pelvis showing cholelithiasis with no biliary dilatation.

Norepinephrine was stopped due to its chronotropic properties, while vasopressin and phenylephrine were continued [1]. Blood cultures were sent, and broad-spectrum intravenous antibiotics were administered, including cefepime, vancomycin, and metronidazole. The ensuing blood cultures showed Gram-negative coccobacilli, and the patient was transferred to the medical intensive care unit (MICU) for further management.

Initial lumber puncture was attempted but failed and repeat lumber puncture was done after days on broad-spectrum antibiotics using fluoroscopic intervention by interventional neurology which was significant for the CSF findings shown in Table 2.

**Table 2:** CSF findings CSF: Cerebrospinal fluid; VDRL: venereal disease research laboratory

Test	Results	Reference Value
CSF analysis		
CSF appearance	Clear	Clear
CSF glucose	128 mg/dL	40 - 70 mg/dL
CSF protein	129 mg/dL	12 - 60 mg/dL
VDRL CSF	Non-reactive	Non-reactive
CSF color	Xanthochromia	Clear
CSF cryptococcal antigen	negative	Negative
CSF culture	No growth at five days	No growth at five days
Red blood cells, CSF	2.0	<=0.0 cells/uL
White blood cells CSF	4.0	<=5.0 cells/uL

**Figure 1:**
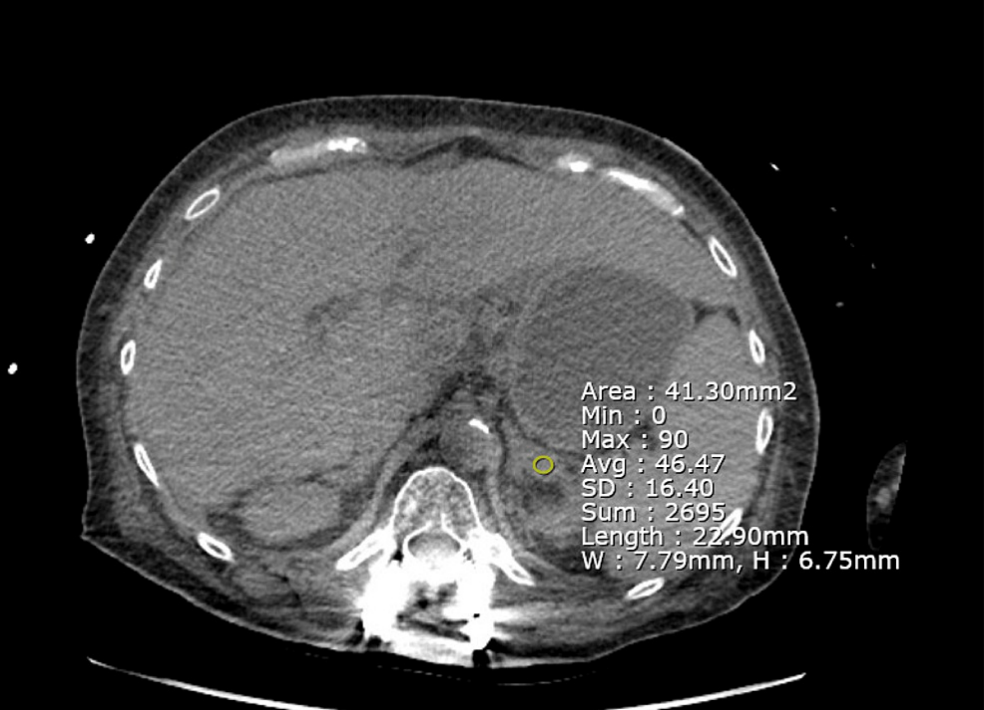
Bilateral adrenal densities attributed to adrenal hemorrhage. Hounsfield units of 46.47 can be appreciated which is likely to be secondary to the hematoma inside of the adrenal gland.

**Figure 2:**
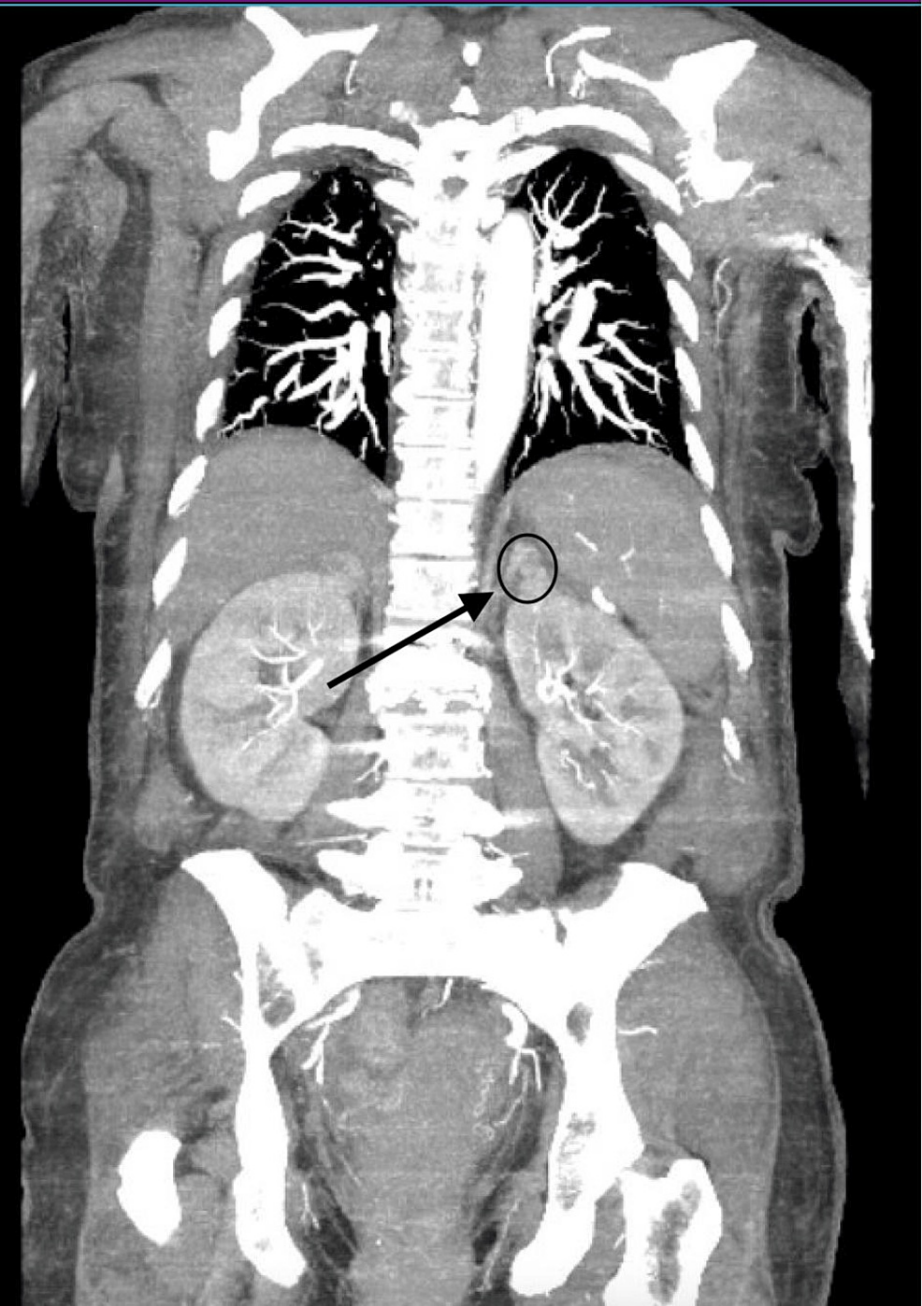
Adrenal hemorrhage/mass "black arrow pointing at a black circle"

The CT scan of the abdomen and pelvis was reevaluated and further disclosed a minor bilateral adrenal hemorrhage with Hounsfield units of 46.47, which heightened the suspicion of WFS (Figure 1 and Figure 2).

In the MICU, the patient was weaned off vasopressors, passed a spontaneous breathing trail, and was extubated and started on stress steroids with hydrocortisone 50 mg q6 and fludrocortisone 0.05mg; the patient's blood pressure progressively improved, and the patient was transferred to the regular medical floor.

Patient's blood culture showed Neisseria meningitidis and the antibiotic regimen was changed to ceftriaxone 2g daily, the patient finished antibiotic treatment and was discharged after the liver function test normalized initially being elevated likely secondary to hypotension.

## Discussion

WFS is a rare but potentially fatal condition associated with meningococcal septicemia. Despite the advent of new microbials, meningococcal infection remains a leading cause of morbidity and mortality [2-6]. Gram staining of the CSF is still considered an important method for rapid diagnosis, but culture from CSF or skin lesion biopsy is the gold standard [2-8].

Disseminated intravascular coagulation may affect adrenal glands causing WFS. Most of the diseases caused by meningococci occur in children under two years of age, but they can occur at any age [9]. In this case, early diagnostic lumbar puncture failed due to a difficult site in view of previous laminectomy, and a successful one was done by interventional neurology after antibiotics of a wide spectrum were administered, falsely making the results negative. However, blood cultures can be an alternative way of diagnosis. Gram stain was positive for Gram-negative coccobacilli, and blood culture later came positive for Neisseria meningitidis. In our patient, the final diagnosis was established by positive blood cultures and adrenal hemorrhage on CT imaging of the abdomen and pelvis.

One of the first presentation signs in this patient was ventricular tachycardia with hemodynamic instability requiring multiple shocks and amiodarone infusion. Primary presentation with ventricular tachycardia indicated possible underlying cardiac issues leading to cardiogenic shock. Transthoracic echocardiogram showed a normal ejection fraction (50%) without significant valvular abnormalities. Meanwhile, elevated WBC and lactic acidosis make diagnosis of septic shock more likely, later being supported by blood culture results. As shown by previous studies, patients with moderate to severe sepsis have a greater likelihood of exhibiting various cardiac arrhythmias [5,6]. The majority of these clinical studies were focused on the diagnosis, management, predictors, and outcomes of new-onset AF in the general intensive care patient population [9]. Studies providing an assessment of atrial and ventricular arrhythmias are currently lacking. The patient was also started on an intravenous infusion of norepinephrine, which could be another cause of ventricular tachycardia in this patient. Association of ventricular arrhythmias was also shown in the use of vasopressors, but controlled data on proarrhythmic potential in vasopressors is lacking. The Society for Critical Care Medicine released a guideline in 2017 for the diagnosis of critical illness-related corticosteroid insufficiency (CIRCI), suggesting a random serum cortisol level of less than 10 µg/dL for a diagnostic threshold to consider steroid replacement [10].

Stress doses of corticosteroids were started empirically; the morning cortisol level was obtained later and was 7.9 mcg/dl supporting initiated management. Proper treatment allowed patient survival with a sufficient recovery level to be discharged home.

## Conclusions

WFS remains challenging to diagnose, especially with other multiple comorbidities and a variety of presentations. Timely suspicion with appropriate diagnostic evaluation and aggressive management, including administration of antibiotics and corticosteroid replacement, should be immediately started to improve patient outcomes.

The diagnosis of acute adrenal insufficiency in critical illness remains challenging, especially with other forms of shock complicating the picture.
